# 546. A Retrospective Review of Biosynthetic Wound Matrix in Management of the Split Thickness Donor Site

**DOI:** 10.1093/jbcr/irag033.243

**Published:** 2026-04-06

**Authors:** Arpana Jain, Anesh Prasai, Suzanne C Osborn, Philomene Spadafore, Erica Whetten, Karen J Richey, Kevin N Foster

**Affiliations:** Diane & Bruce Halle Arizona Burn Center - Valleywise Health, Phoenix, AZ; Avita Medical, Denver, CO; Diane & Bruce Halle Arizona Burn Center - Valleywise Health, Phoenix, AZ; Diane & Bruce Halle Arizona Burn Center - Valleywise Health, Phoenix, AZ; Valleywise Health Medical Center, Phoenix, AZ; Diane & Bruce Halle Arizona Burn Center - Valleywise Health, Phoenix, AZ; Diane & Bruce Halle Arizona Burn Center - Valleywise Health, Phoenix, AZ; Diane & Bruce Halle Arizona Burn Center - Valleywise Health, Phoenix, AZ

## Abstract

**Introduction:**

The split-thickness skin grafting (STSG) donor site creates a secondary partial-thickness wound that is quite painful, prone to complications, and may require up to three weeks to heal. Current management often leads to frequent dressing changes, excessive exudate, and prolonged hospital stays. Biosynthetic wound matrices (BWM) may improve outcomes by reducing pain, minimizing dressing changes, and expediting healing, thereby decreasing length of stay and opioid use. This retrospective study evaluates the feasibility and effectiveness of BWM in donor-site management while providing preliminary data for future prospective trials.

**Methods:**

This retrospective, single-arm cohort study was conducted at a regional burn center (June 2024–June 2025) under IRB approval. All patients undergoing STSG with donor sites managed using a biosynthetic wound matrix (BWM) were included. The primary outcomes were time to complete epithelialization, the number of dressing changes, and hospital length of stay. Secondary outcomes included donor-site infection, non-healing, and readmission. Descriptive statistics and subgroup comparisons (inpatient vs outpatient, adult vs pediatric) were performed.

**Results:**

A total of 21 donor events were analyzed, with a median patient age of 42 years (range, 2–80 years), 67% male, and 48% inpatients. The time to complete epithelialization (TCE) was available for 17 sites, with a median of 20 days (IQR, 14–29; range, 9–48). Outpatients (n = 11, TCE available in 7) epithelialized more rapidly than inpatients (n = 10, TCE available in 10), with median times of 14 days vs 25.5 days, respectively. No donor-site infections, non-healing wounds, or readmissions were documented among evaluable cases; 4 patients were lost to follow-up. The median length of stay was 7.5 days (IQR 5–9) for inpatients and 0 days for outpatients. Analgesic and pain data were not complete in this dataset and are undergoing separate analysis.

**Conclusions:**

The use of a BWM for split-thickness skin graft donor sites was associated with reliable epithelialization within 2–3 weeks, a low burden of dressing changes, and no documented donor site infections, non-healing of wounds, or readmissions, both in inpatient and outpatient settings.

**Applicability of Research to Practice:**

By reducing resource utilization and eliminating donor-site morbidity as a driver of hospital stay, BWM provides a practical approach that can simplify donor-site management and improve patient comfort across burn centers. A future prospective study may reveal a standardized approach to the donor site management.

**Funding for the study:**

AVITA Medical.

